# Unbalanced historical phenotypic data from seed regeneration of a barley *ex situ* collection

**DOI:** 10.1038/sdata.2018.278

**Published:** 2018-12-04

**Authors:** Maria Y. Gonzalez, Stephan Weise, Yusheng Zhao, Norman Philipp, Daniel Arend, Andreas Börner, Markus Oppermann, Andreas Graner, Jochen C. Reif, Albert W. Schulthess

**Affiliations:** 1 Department of Breeding Research, Leibniz Institute of Plant Genetics and Crop Plant Research (IPK), D-06466, Gatersleben, Germany; 2 Department of Genebank, Leibniz Institute of Plant Genetics and Crop Plant Research (IPK), D-06466, Gatersleben, Germany

**Keywords:** Plant sciences, Agricultural genetics, Plant breeding

## Abstract

The scarce knowledge on phenotypic characterization restricts the usage of genetic diversity of plant genetic resources in research and breeding. We describe original and ready-to-use processed data for approximately 60% of ~22,000 barley accessions hosted at the Federal *ex situ* Genebank for Agricultural and Horticultural Plant Species. The dataset gathers records for three traits with agronomic relevance: flowering time, plant height and thousand grain weight. This information was collected for seven decades for winter and spring barley during the seed regeneration routine. The curated data represent a source for research on genetics and genomics of adaptive and yield related traits in cereals due to the importance of barley as model organism. This data could be used to predict the performance of non-phenotyped individuals in other collections through genomic prediction. Moreover, the dataset empowers the utilization of phenotypic diversity of genetic resources for crop improvement.

## Background & Summary

Cereals are staple food and a valuable source of nutrients around the world^1^. Among them, barley (*Hordeum vulgare* sp.) is the fourth most produced crop^2^. The main end-uses of barley are brewing, feed, and food production^3^. In terms of crop adaptation barley can be classified into two distinct gene pools: winter and spring type^4–6^. While winter type barley needs vernalization for flowering stimulation, spring type barley does not require it^3^. Barley has a diploid genome and its 7 chromosomes represent the base genome of all Triticeae species. For this and many more reasons barley has become a model organism in cereal genetics and genomics^7^. In addition, the availability of a high quality reference sequence of the barley genome, well established protocols for genome editing and elaborated approaches for genomic selection will greatly benefit barley breeding in the future^7–11^.

Establishing germplasm collections has involved assemblage and preservation of the existing allelic diversity and their utilization^12,13^. In the case of barley, more than seven decades of major efforts have resulted in about half a million *ex situ* accessions worldwide^13–15^. Germplasm collections are an outstanding resource of genetic diversity for research and plant improvement. For instance, genebank collections represent a rich source of unexplored trait variation which is absent in public and private breeding programs. This variation could potentially boost selection gain in plant breeding to increase both yield potential and sustainability and to facilitate adaptation to global change^16,17^. However, leveraging genetic resources of public germplasm collections is still a challenge due to the lack of phenotypic information and the high investments required for the systematic characterization of plant material^9,18,19^. Recently, a method for the exploitation of germplasm based on genomics was proposed^19^. In this context, genebanks are encouraged to maximize the reuse of both phenotypic and genotypic data by the implementation of the FAIR principles referring to: Findability, Accessibility, Interoperability, and Reusability^20^. For example, historical phenotypic records for traits with agronomical relevance have been accumulated during the seed regeneration process at genebanks but are not publicly available or the access to them is limited^16,19–23^.

This study presents original and ready-to-use processed phenotypic data with the aim of leveraging the use of historical information collected during seed regeneration. The data correspond to historical records on traits flowering time (FT), plant height (PH), and thousand grain weight (TGW) accumulated for seven decades plus the outlier status of all data points and the Best Linear Unbiased Estimations (BLUEs) for winter and spring barley accessions pertaining to these traits. This historical information belongs to the barley collection of the Federal *ex situ* Genebank for Agricultural and Horticultural Plant Species hosted at the Leibniz Institute of Plant Genetics and Crop Plant Research (IPK) in Gatersleben (Germany). Conserving and managing a total of ~22,000 accessions, the IPK Genebank manages the sixth largest collection worldwide, which covers a broad range of phenotypic variation^14,15,24,25^. This data publication complements a previous research publication^25^ which focuses on the valorization of genetic resources by developing, validating and employing a curated data set from seed regeneration. Moreover, part of these BLUEs was recently used to show the potetial of genome wide association for FT in genebank materials of spring barley^26^.

## Methods

### Plant material

The barley collection at the IPK amounts to ~22,000 accessions. These accessions were assembled by means of worldwide collecting expeditions, seed exchange with other institutes, and donations. Accession-related information is being documented in the genebank information system of the IPK (GBIS)^22^. This study includes FT, PH, and TGW data recorded during seed regeneration for approximatelly 60% of the barley accessions.

### Seed regeneration produced an unbalanced historical data source

**Figure 1:**
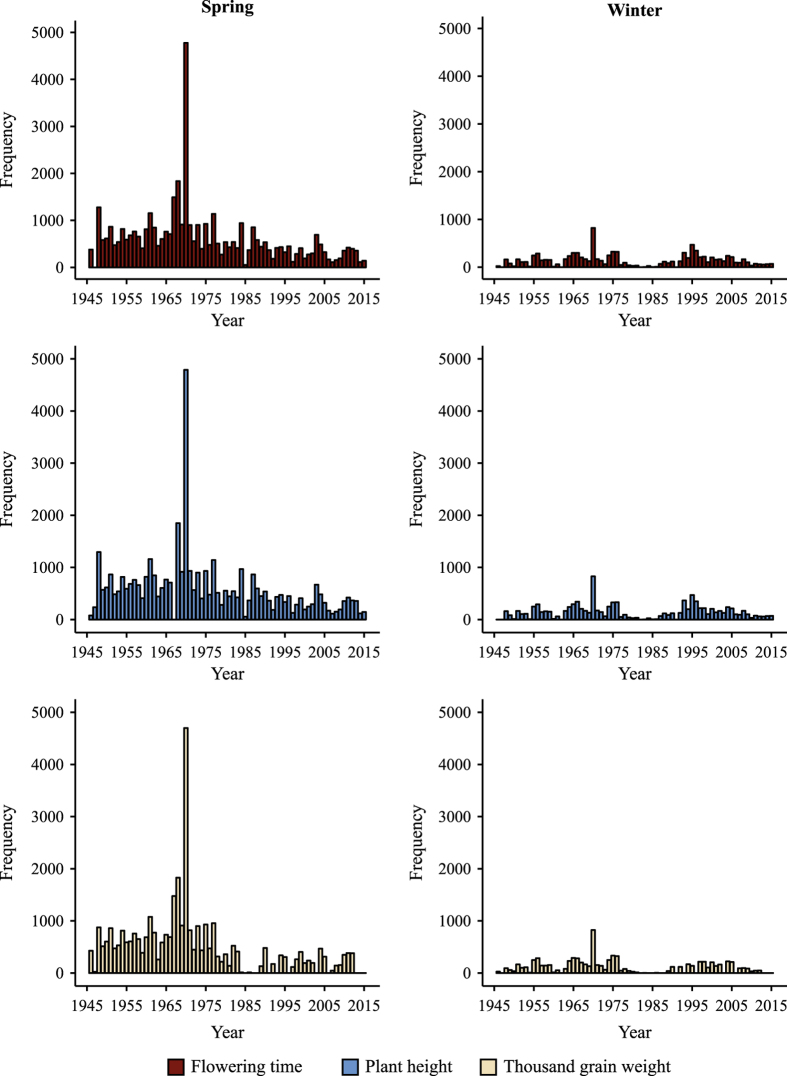
Number of accessions tested for flowering time (FT), plant height (PH), and thousand grain weight (TGW) for the time period from 1946 until 2015 for spring (left) and winter barley (right).

Seed regeneration is aimed to supply seed requirements for (i) safeguarding the stored genetic diversity when sample size and seed viability drop beneath a pre-stablished treshold, (ii) conserving new genotypes within the genebank, (iii) research, and (iv) fulfilling external demands of germplasm^27^. The seed regeneration routine in the genebank generated non-orthogonal phenotypic data^23,28,29^ across traits and years, e.g., only 12 accessions were evaluated for TGW in 1984 while a record number of 4,789 accessions were characterized in 1970 for PH. Additionally, there were 1% of cases when accessions were multiplied more than once in a year. One of the reasons for this was, for instance, the need to check whether the plant material required vernalization or not. Moreover, the introduction of cold storage in the year 1976 abruptly decreased the periodicity of data generation during seed regeneration, because storage time switched from ~3 to >20 years^27^ . Furthermore, the use of the collection, or parts of it, in research projects had a positive impact in the amount of data collected per year. For example, the protein screening of cereal genetic resources carried in 1970 brought the largest number of regenerated accessions in a single year (Fig. 1). The data of the present study is based on seed regenerations during the 1946–2015 period. Seed regeneration for barley was conducted in Gatersleben since 1946 in different seasons according to the growth habit of accessions. In more detail, winter accessions were planted between September and December while spring accessions were sown from February until April.

### Traits assessed on seed germplasm regeneration

Each accession was multiplied using plots of at least 3 m^2^ and traits FT, PH, and TGW were assessed during seed regeneration. FT stands for the number of days when 50% of the plants reached flowering. For winter barley, FT is expressed in days after the 1^st^ of January of each year. For spring barley, FT was expressed in days after the sowing date. PH was assessed in cm from the soil surface to the top of spike including awns. TGW was determined after seed harvest and expressed in g on a ~12.5% grain moisture basis. Seeds were harvested at maturity stage and were temporary stored at room temperature. Before the 2005/2006 season the standard protocol for TGW assessment at the genebank was based on the average weight of three samples, each containing 100 grains, which was then extrapolated to 1000 grains. From the 2005/2006 season onwards TGW has been determined by using an automatic Marvin digital seed analyzer and considering a seed sample with up to 100 grains. The data management at the genebank was manual until 2011. In this sense, the information was first recorded in field books, then transferred to card files and lately digitized for data storage and computational analysis. From 2011 onwards Personal Digital Assistants (PDAs) were used.

## Methods for data processing

### Statistical model

No formal field experimental design was used during seed regeneration while the dataset contains only 1% of cases when accessions were evaluated more than once in a year. For this reason, an unreplicated completely randomized experimental design was assumed for each regeneration cycle during data processing. According to the assumed design, the experimental unit corresponded to a plot. Phenotypic data of each barley type were analyzed separately based on the following mixed model:

(1)Trait∼µ+Genotypes+Years+Error,

where μ is the population mean and “Genotypes” were the genetic effects of accessions, which were assumed as fixed factors, while years and error were treated as random. Variances of errors were modelled as specific for each year. In a first step, Equation (1) was used for outlier detection. Later, the BLUEs of accessions were computed by re-fitting the model in Equation (1) but using and enhanced historical dataset in which data points detected as outliers during the first step were discarded.

### Code availability

Mixed model equations were solved using the Restricted Maximum Likelihood (REML) algorithm as implemented in ASReml-R^30^. All described statistical approaches were performed in R environment (Version 2.15.3)^31^. Scripts used for outlier detection and estimating BLUEs are included together with the dataset in the public repository described below (Data Citation 1). The use of the code requires the download of the datasets, save them in a working directory and set the working directory in the scripts. The scripts run for a single trait according to one growth habit. For instance, the example scripts run for flowering time (FT) for spring barley. In this case, the resulting files are labeled as “Data.corrected.FT.txt” or “BLUEs.FT.txt” for outlier detection and estimating BLUEs, respectively. In this regard, this study involves 12 outputs that were compiled in four files which are described below.

## Data records

The data compiled for this study is publicily available in the Plant Genomics and Phenomics Research Data Repository (PGP) (http://edal-pgp.ipk-gatersleben.de/)^32^ and can be accessed here as (Data Citation 1). The dataset is formated using the ISA-Tab format^33^ to guarantee a uniform and easy-readable semantical description. It contains the original data as well as the processed data. While the investigation file describes the general project information, the two study files (“s_Spring_Barley.txt” and “s_Winter_Barley.txt”) provide information about the investigated accessions. They contain information such as: (i) accession identifiers, e.g., the accession ID as an unique and stable database generated code at the genebank and accession number wich is typically used for researchers but is not stable over the time, (ii) sowing_date corresponding to day.month.year, (iii) harvest_year, (iv) country as geographic place of collection reported by donors or collectors, and (v) the comment column which shows two groups of accessions whose countries are mentioned in the manuscript as Germany and Soviet Union. In this regard, the group Germany includes accessions from Germany and [Former] East Germany. The group Soviet Union stands for accessions from [Former] Union of Soviet Socialist Republics, Armenia, Azerbaijan, Belarus, Georgia, Estonia, Kyrgystan, Latvia, Lithuania, Moldova, Russia, Tajikistan, Turkmenistan, Ukraine and Uzbekistan. Furthermore, some modifications were done with respect to the original data, e.g. the harvest year 1946 contained only 2 records for PH in winter type barley, which caused serious convergency problems during the fitting of mixed models. For this reason, these two datapoints were removed from the PH records of winter barley.

**Figure 2:**
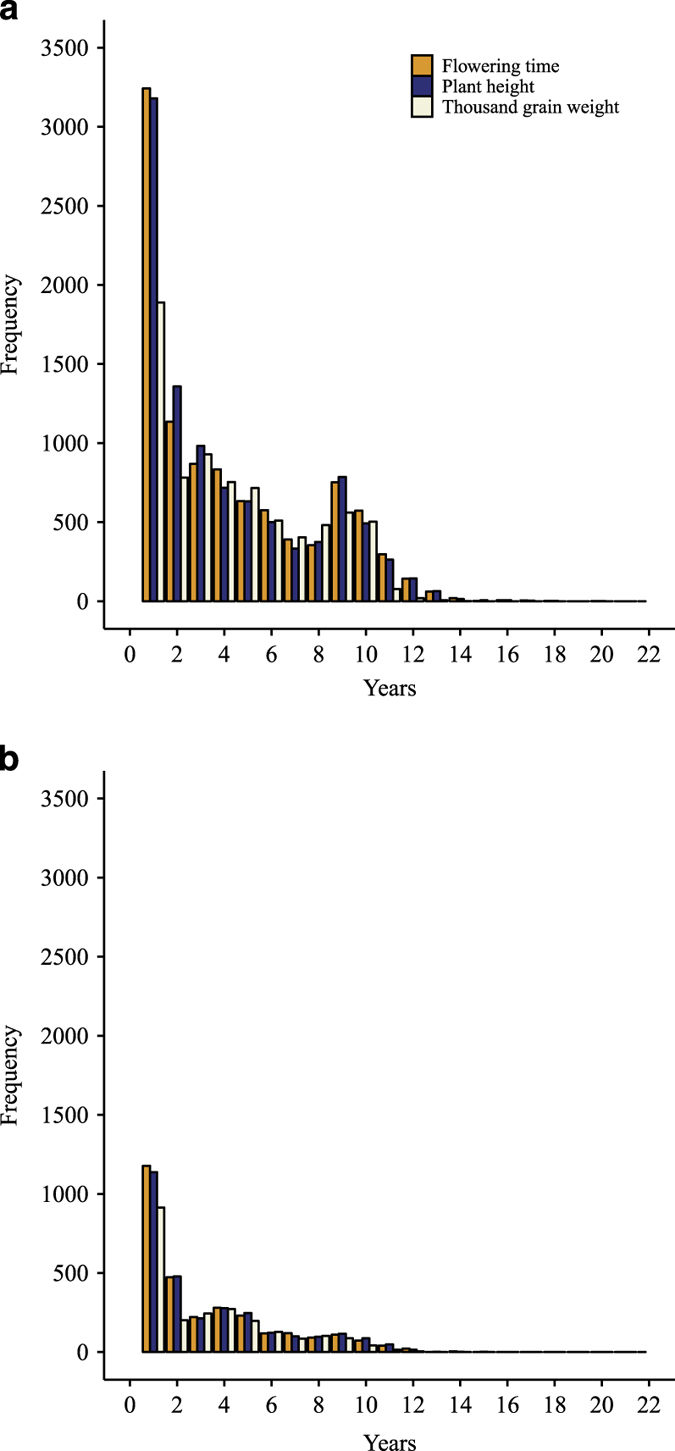
Number of years in which accessions were tested for flowering time (FT), plant height (PH), and thousand grain weight (TGW). (**a**) Spring, and (**b**) winter barley growth habits.

**Figure 3:**
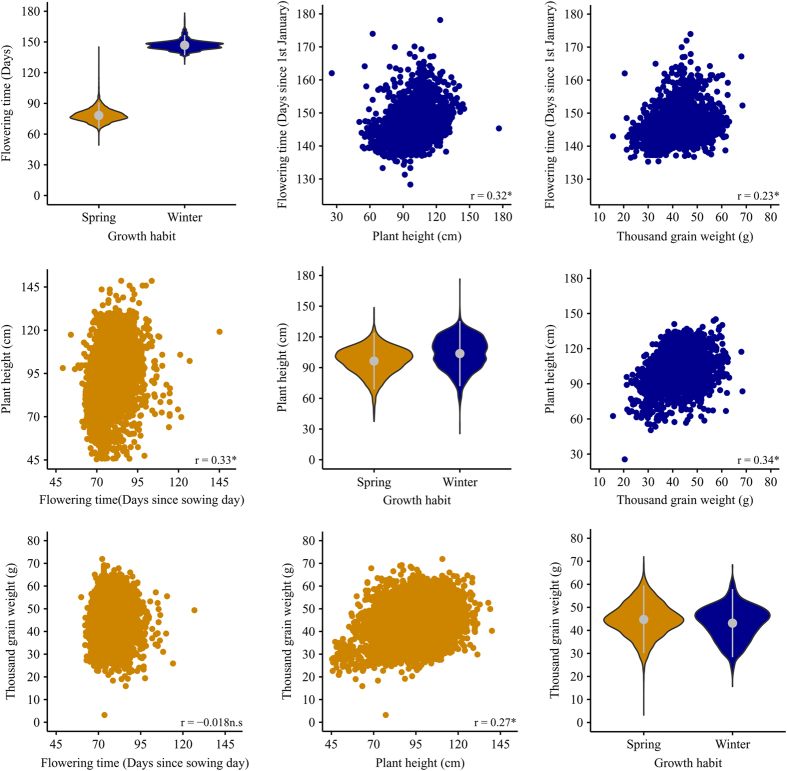
Matrix plot of dispersion and relationships among Best Linear Unbiased Estimators (BLUEs) of accessions for flowering time (FT), plant height (PH), and thousand grain weight (TGW). Winter and spring barley are represented in blue and yellow colors, respectively. The diagonal shows the dispersion for each trait as violin plots whereas the upper and lower triangle stands for the relationships among traits. The Pearson’s correlation coefficient (*r*) is presented in the lower right corner of every plot for each trait combination. Correlations that significantly differ from zero (p-value < 0.0001) are indicated with * while those being not significant are indicated with n.s.

The assay files of the present study were separated in the historical phenotypic data (“a_Historical.Data_Spring.txt” and “a_Historical.Data_Winter.txt”), which was provided from the IPK genebank information system and was first screened for outliers. Then, outliers were excluded to produce the enhanced assay files (“a_Enhanced_Historical.Data_Spring.txt” and “a_Enhanced_Historical.Data_Winter.txt”). These files accomodated records for up to 2,967 and 9,898 winter and spring accessions, respectively (Table 1). Each accession was phenotyped from 1 to 22 years (Fig. 2) and in each year a range from 12 to 4,789 accessions, across traits, were evaluated (Fig. 1). The heritability for all traits was high and it increased further by up to 17% when applying an outlier correction^25^ (Table 2). The Pearson’s correlation coefficient (*r*) estimated on the enhanced data for pairs of years with at least 50 overlapping accessions ranged from 0.60 to 0.72 (Table 3). The precision in computing the BLUEs amounted to 0.89 for TGW and 0.85 for both FT and PH, respectively^25^. Moreover, the maximum coefficient of variation of the year on the enhanced data set was 0.22 (Table 4). Ninety percent of these genetic resources were collected or originated from 30 geographic places. Ethiopia with 32.1% of accessions was a predominant origin for spring barley followed by 7.2% from Germany. Interestingly, although 12.4% of winter barley accessions were collected or originated from the Soviet Union, there was not a clear predominant place of collection for this type of barley which was reflected by a more uniform frequency distribution of accessions according to collection places (Table 5). Furthermore, the dataset contains an additional folder with the BLUEs of accessions included in the files “BLUEs_Spring.txt” and “BLUEs_Winter.txt” (Fig. 3), that were estimated based on the enhanced historical data files. The corresponding study files are labeled as “s_Spring_Barley.txt” and “s_Winter_Barley.txt”.

## Technical Validation

Validation involves outlier detection, bias assesment for first and second degree statistics and validation of BLUEs of accessions. Methods, results and discussion of this strategy were described in a previous research publication^25^. However, here we make a brief description of validation methods.

### Enhancing the quality of the historical data set by implementing an outlier detection approach

Outliers may jeopardize the quality of the data negatively affecting statistical estimates^34,35^. The presence of outliers in the historical dataset (Data Citation 1) is plausible because the data was assembled for seven decades under fluctuating conditions of data and seed regeneration management, as well as contrasting weather conditions across years, among others. Both, the assessment and management of outliers in unbalanced historical datasets are challenging. We used an outlier inspection approach by combining re-scaled median absolute deviation of standardized residuals with a Bonferroni-Holm test to flag data points as outliers^35^. A data-point was declared as outlier by the implemented test according to a predefined significance threshold of p-value < 0.05. We removed the outliers from the historical data set to obtain an enhanced historical dataset (Data Citation 1). Considering genotypes and years as random effects, Equation (1) was re-fitted in order to check the impact of outlier exclusion on variance components and heritability. Heritability was computed as follows: $h^{2}=\frac{{\hat{\sigma }}_{G}^{2}}{{\hat{\sigma }}_{G}^{2}+\frac{{\hat{\sigma }}_{e}^{2}}{\bar{Y}}}$, where ${\hat{\sigma }}_{G}^{2}$ denotes the estimator of the genetic variance, ${\hat{\sigma }}_{e}^{2}$ corresponds to the average variance estimated for the errors, and $\bar{Y}$ stands for the average number of years when genotypes have been tested. Assuming random genotype and fixed year effects on Equation (1), the coefficient of variation of the year was computed as:$=\frac{\sqrt{{\hat{\sigma }}_{e}^{2}}}{YE}$, where ${\hat{\sigma }}_{e}^{2}$ corresponds to the year-specific error variance and YE refers to the year effect.

### Studying the potential bias in estimating first- and second-degree statistics for different missing data scenarios

On average, seed regeneration activities before 1976 were carried out every 3 years for each accession. This was mainly because seed storage was formerly performed at room temperature^27^. However, this condition led to evaluate blocks of accessions corresponding to the year when they entered the genebank, which is often reflecting specific collection hotspots. Therefore, the missing value structure of the phenotypic data collected is potentially deviated from the random scenario. Since estimating first and second degree statistics is potentially biased by the missing data structure, a resampling study was performed considering three missing data scenarios. Firstly, a balanced dataset was derived from the enhanced historical dataset of spring barley. This balanced set included phenotypic records for FT and PH available for the years 1948, 1951, 1954, 1957, 1961, and 1970 for 400 spring accessions. These accessions were collected in 10 geographic places: Turkey (99), Greece (91), Germany (56), United States of America (49), Bulgaria (36), Sweden (18), Japan (14), Albania (13), Austria (12), and countries of the former Soviet Union (12). Later, the balanced dataset was sampled based on three missing data scenarios as follows: in Scenario 1, phenotypic records were randomly sampled from three out of six test years for each accession, which amounted to 1,200 phenotypic data points in total. In Scenario 2, the 400 accessions were randomly grouped into 10 clusters and the phenotypic data for each group was randomly subsampled from 3 years gathering 1,200 phenotypic data points in total. In Scenario 3 the 10 places of collection were considered as groups of accessions and phenotypic data from 3 years was randomly subsampled for each group resulting in 1,200 phenotypic data points. Each scenario was sampled 100 times.

Biases in estimating variances of genotypes and errors were calculated as $\frac{\hat{d}-d}{d}$, where $\hat{d}$ stands for the estimated parameters in each sampling run and *d* corresponds to the parameter estimated from the balanced dataset. Moreover, we performed a linear regression of the BLUEs computed for each of 100 resampling runs on the BLUEs from the balanced data set. In this respect, the intercept, the slope, and the coefficient of determination of the linear regression model were considered to measure bias.

### Resampling procedure for assessing the precision in computing BLUEs of accessions

Precise estimates of trait performance are pivotal for decisions makers on research and breeding. Thus, we performed a resampling procedure^36,37^ to assess the precision in estimating BLUEs. The enhanced data set of spring barley was randomly split into two equally sized subsets. Only accessions for which phenotypic data was available in both subsets were considered in each of the 100 resampling runs. Therefore, across 100 runs 3,691, 3,474, and 3,066 accessions were included on average for FT, PH and TGW, respectively. We fitted the model specified in Equation (1) to estimate the BLUEs of accessions in both subsets. Subsequently, precision of estimation was computed as the correlation of BLUEs of accessions between subsets.

## Usage Notes

Maximizing the use of genetic resources will benefit current and future efforts to breed new cultivars that are required to address needs in food security, climate resilience, and sustainability^16,38,39^. However, restricted resources limit the systematic phenotyping of germplasm collections^9,18,19^. The strategy described here is based on data that was routinely collected by curators during seed multiplication cycles and is embedded in the scripts used for outlier detection and BLUEs computation. The scripts run for a single trait according to one growth habit. This strategy could be adapted to other genebanks for the validation of their own data in order to increase the amount of data for well characterized accessions at no extra cost. The value of the data will be further leveraged by genotypic information which will become publicly available soon for the IPK barley collection. In the future, both, phenotypic and genotypic information will facilitate the implementation of genomic prediction which is expected to further boost the utilization of genetic resources for research and breeding^19,40–42^. By providing the investigated data using the ISA-Tab format and publishing them via DOI, all research data and the presented results are available in a FAIR-way^20^ and can be easily re-used.

## Additional information

**How to cite this article**: Gonzalez, M. Y. *et al*. Unbalanced historical phenotypic data from seed regeneration of a barley *ex situ* collection. *Sci. Data*. 5:180278 doi: 10.1038/sdata.2018.278 (2018).

**Publisher’s note**: Springer Nature remains neutral with regard to jurisdictional claims in published maps and institutional affiliations.

## Supplementary Material



## Figures and Tables

**Table 1:** Number of phenotypic records and accessions tested for flowering time (FT), plant height (PH), and thousand grain weight (TGW) for winter and spring barley assessed for the time period from 1946 until 2015.

Trait	Type	No. of accessions	No. of Phenotypic records	No. of outliers removed
Flowering time (days)	Spring	9,898	43,264	550
	Winter	2,967	10,100	270
Plant height (cm)	Spring	9,858	41,933	52
	Winter	2,946	10,238	42
Thousand grain weight (g)	Spring	7,634	33,854	144
	Winter	2,293	7,748	48

**Table 2:** Estimates on historical data and enhanced historical data sets for variance components of genotypes (${\hat{\sigma }}_{G}^{2}$), years (${\hat{\sigma }}_{y}^{2}$), and errors (${\hat{\sigma }}_{e}^{2}$); number of environments (*E*) and heritability (*h*^2^) for flowering time (FT), plant height (PH), and thousand grain weight (TGW) of up to 2,967 winter and up to 9,898 spring barley accessions evaluated in up to 69 years of seed regeneration^25^.

Trait	Spring type		Winter type	
	Historical Data	Enhanced Historical Data	Historical Data	Enhanced Historical Data
	Flowering Time			
${\hat{\sigma }}_{G}^{2}$	24.9	27.62	10.57	12.83
${\hat{\sigma }}_{y}^{2}$	48.1	48.7	73.72	71.32
${\hat{\sigma }}_{e}^{2}$	24.82	16.08	15.32	9.13
*E*	4.42	4.37	3.49	3.4
*h*^2^	0.82	0.88	0.71	0.83
	Plant height			
${\hat{\sigma }}_{G}^{2}$	133.24	134.97	156.75	161.52
${\hat{\sigma }}_{y}^{2}$	116.39	116.5	232.4	233.7
${\hat{\sigma }}_{e}^{2}$	95.76	91.57	90.53	84.71
*E*	4.26	4.25	3.49	3.47
*h*^2^	0.86	0.86	0.86	0.87
	Thousand grain weight			
${\hat{\sigma }}_{G}^{2}$	44.23	45.19	37.79	39.68
${\hat{\sigma }}_{y}^{2}$	17.85	17.73	17.51	17.27
${\hat{\sigma }}_{e}^{2}$	18.13	16.38	14.01	10.97
*E*	4.45	4.43	3.4	3.38
*h*^2^	0.92	0.92	0.9	0.92

**Table 3:** Pearson’s correlation coefficient (*r*) estimated between pairs of years with at least 50 overlapping accessions for the time period from 1946 until 2015 for spring and winter barley.

Trait	Spring type		Winter type	
	Coefficient of correlation (*r*)	No. of pairs of years	Coefficient of correlation (*r*)	No. of years pairs
Flowering time	0.65	610	0.64	118
Plant height	0.60	576	0.60	128
Thousand Grain Weight	0.72	407	0.70	89

**Table 4:** Mean ± standard deviation (SD) and range for the coefficient of variation of the residuals calculated for each year for flowering time, plant height, and thousand grain weight for spring and winter barley on the enhanced dataset.

Trait	Spring type		Winter type	
	Mean±SD	Range	Mean ± SD	Range
Flowering time	0.049 ± 0.013	0.02–0.086	0.019 ± 0.006	0.007–0.035
Plant height	0.099 ± 0.018	0.064–0.157	0.087 ± 0.028	0.029–0.225
Thousand grain weight	0.090 ± 0.018	0.060–0.134	0.073 ± 0.021	0.00006–0.12

**Table 5:** Distribution of spring and winter barley by geographic place of collection reported by donors or collectors (origin).

Winter			Spring		
Origin	No. of accessions	Percentage	Origin	No. of accessions	Percentage
Soviet Union	374	12.6	Ethiopia	3,174	32.1
Turkey	349	11.8	Germany	717	7.2
Germany	324	11.0	Turkey	637	6.4
Japan	254	8.6	Unknown	536	5.4
Korea	247	8.3	Soviet Union	359	3.6
Unknown	185	6.2	India	346	3.5
United States of America	138	4.7	United States of America	340	3.4
French Republic	110	3.7	Nepal	328	3.3
China	108	3.6	China	319	3.2
India	73	2.5	Greece	260	2.6
Greece	66	2.2	Japan	227	2.3
Canada	64	2.2	Iran	219	2.2
United Kingdom	64	2.2	Italy	200	2.0
Italy	61	2.1	Israel	198	2.0
Bulgaria	53	1.8	Pakistan	186	1.9
Syria	50	1.7	Austria	139	1.4
Afghanistan	40	1.3	Afghanistan	127	1.3
Romania	40	1.3	Libya	122	1.2
Poland	36	1.2	Slovak Republic	101	1.0
Hungary	34	1.1	French Republic	98	1.0
Switzerland	32	1.1	Sweden	97	1.0
Ethiopia	30	1.0	Poland	91	0.9
Others (21 origins)	234	7.9	Others (48 origins)	1,077	10.9
Total	2,967	100	Total	9,898	100
